# On-chip MIC by Combining Concentration Gradient Generator and Flanged Chamber Arrays

**DOI:** 10.3390/mi11020207

**Published:** 2020-02-17

**Authors:** Xiao-Yan Zhang, Zhe-Yu Li, Kose Ueno, Hiroaki Misawa, Nan-Qi Ren, Kai Sun

**Affiliations:** 1 State Key Laboratory of Urban Water Resource and Environment, School of Environment, Harbin Institute of Technology, Harbin 150090, China; xyzhang774985529@163.com (X.-Y.Z.); rnq@hit.edu.cn (N.-Q.R.); 2 Research Institute for Electronic Science, Hokkaido University, Sapporo 001-0021, Japan; k-ueno@es.hokudai.ac.jp (K.U.); misawa@es.hokudai.ac.jp (H.M.); 3 Department of Applied Chemistry & Institute of Molecular Science, National Chiao Tung University, Hsinchu 30010, Taiwan

**Keywords:** minimum inhibition concentration (MIC), microfluidic, bacteria, antibiotics

## Abstract

Minimum inhibition concentration (MIC) of antibiotic is an effective value to ascertain the agent and minimum dosage of inhibiting bacterial growth. However, current techniques to determine MIC are labor intensive and time-consuming, and require skilled operator and high initial concentration of bacteria. To simplify the operation and reduce the time of inhibition test, we developed a microfluidic system, containing a concentration generator and sub-micro-liter chambers, for rapid bacterial growth and inhibition test. To improve the mixing effect, a micropillar array in honeycomb-structure channels is designed, so the steady concentration gradient of amoxicillin can be generated. The flanged chambers are used to culture bacteria under the condition of continuous flow and the medium of chambers is refreshed constantly, which could supply the sufficient nutrient for bacteria growth and take away the metabolite. Based on the microfluidic platform, the bacterial growth with antibiotic inhibition on chip can be quantitatively measured and MIC can be obtained within six hours using low initial concentration of bacteria. Overall, this microfluidic platform has the potential to provide rapidness and effectiveness to screen bacteria and determine MIC of corresponding antibiotics in clinical therapies.

## 1. Introduction

Antibiotic resistance has been widely believed as one of the worldwide problems to be solved, and also the gravest threats to human health [1]. It may lead to 25,000 deaths every year and cost the European Union €1.5 billion annually [2,3]. The main cause of antibiotic resistance, which is increasingly being recognized, is the overuse and misuse of antibiotics in clinics, agriculture, animal husbandry, and aquaculture [4,5,6,7]. Antibiotic susceptibility testing (AST) is an essential step in preventing the overuse and misuse of antibiotics, by providing the effective antibiotic agents and minimum dosage of inhibiting bacterial growth [8]. Minimum inhibition concentration (MIC) plays an important role in clinics to ascertain the proper drug to patients. However, the manual processes within the general methods to determine MIC of relative antibiotics, have severe negative influence on the throughput and process efficiency. By shaking flasks or plate counts, the batch culture of the bacteria requires over 24 h to obtain an outcome. The time-consuming process results in high patient mortality. Therefore, rapid bacterial growth inhibition assay is extremely important. Instead of batch culture, microfluidics can serve as a powerful tool for MIC because of its advances in miniaturization, integration, high throughput analysis, controllable conditions, and low sample consumption [9,10,11]. Microfluidic technology has been an effective method for bioanalysis, such as label-free cell separation, single-cell biophysics, capturing agents for specific bioseparation [12,13,14,15,16]. Moreover, microfluidics is capable of confining individual bacteria to a micrometer scale environment to enable quick bacterial growth [17,18], because the bacteria tend to accumulate in the surroundings where the space is sufficiently enclosed [19,20]. The methods of confining bacteria into micro scale include agar immobilization, droplet analyzer, and suspension in micro-scale environment. 

Most of bacteria with flagella can swim and they could be knocked off track by the collisions between water and solute molecules, resulting in bacteria moving in random directions. Therefore, it is difficult to observe and record in the process of bacterial growth. One key strategy in dealing with this challenge is to form a monolayer for immobilizing the bacteria between a layer agar and a cover plate in microfluidic devices [21], which also enable optical imaging on the focal plane to observe bacterial growth directly and count bacteria precisely. Li et al. [22] and Choi et al. [23] make use of agar on chip for the observation of bacterial growth and the determination of MIC. Another advantage of agar is its porous structure, which has been widely used to generate concentration gradient in antibiotic inhibition assay. The general method of evaluating the concentration of antibiotics is to introduce Rhodamine B or fluorescein solution and get the fluorescent intensity. Therefore, only antibiotics with the similar molecular weights to the fluorescent dye can be concluded [21,22]. Another issue of this method is the influence due to bacterial chemotactic signaling [23,24], which will enable bacteria to move or grow toward nutrients and away from toxins. When the environmental conditions become hostile, chemotaxis will result in the tendency of low concentration.

Droplet microfluidics has the potential for rapid MIC determination due to single cell confinement in a few picoliter or even femtoliter [25,26,27,28]. By combining optical diffusometry with bead-based immunoassays, Chung et al. developed a multiplexed and time-saving (within 2 h) platform for quantitative observation of *Pseudomonas aeruginosa* co-cultured with *Staphylococcus aureus* and inhibition test with antibiotics [29]. Hydrogel droplets were used to remove the external oil surrounding the aqueous drops, so that the concentration gradient of antibiotics could be generated around the droplet population [30]. Though there are advances in droplet-based microfluidic tools, lack of a generic platform for integration of all necessary operations still makes it difficult for non-specialist labs.

Confining bacteria in micro environment can shorten growth cycle of bacteria. Most methods used nano-scale or sub-nano-scale chambers to culture bacteria [31,32]. However, to ensure the being of bacteria in the per unit volume of the above chambers, initial bacteria concentration has to be no less than 10^6^ cells per mL. If the initial bacterial concentration is lower, there may be possibility that no bacteria was kept or captured in a nanoliter or sub-nanoliter chamber. Therefore, pre-culture is essential, which will elongate the total detection time. However, it has been ignored in most of the published works. Comparing with the above nanoliter chambers, the initial bacterial concentration used in micro-scale could be reduced to 10^3^ cells per mL. Moreover, the oxygen and nutrients are sufficient for bacterial suspension culture in micro-scale chambers, which avoid densely packed core region after the vigorous bacteria growth [33]. With the advantages of shear stresses being controlled, this kind of microfluidic platforms is also used to investigate cell viability, motility, and functionality. Shao et al. cultured endothelial cells under static conditions on microfluidic chip and studied the morphology and cytoskeleton of their reaction to the oscillatory and pulsatile shear stress [34]. 

In this work, we developed a microfluidic chip for bacteria continuous culture, which comprises a submicron-liter-chamber array for bacterial growth and a concentration gradient generator by novel micro mixers. To refresh the medium of chambers, a novel flanged chamber is designed to culture bacteria at the continuous flow. We set up a detection system to monitor bacterial growth by the variation of bacteria concentration and study the bacterial inhibition. Based on the optimized conditions, the bacterial growth with antibiotic inhibition on chip was quantitatively measured. Based on rapid bacterial growth on microfluidic chip, the MIC can be obtained and the bacterial growth inhibited by antibiotic on chip was quantitatively measured.

## 2. Materials and Methods 

### 2.1. Chip Design and Fabrication

**Figure 1:**
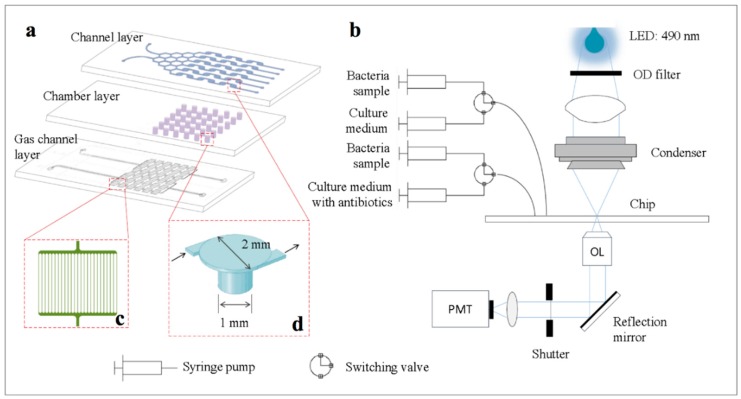
Schematic illustrations of chip structures and microfluidic system. (**a**) Illustration for three layers of chip: channel layer is used to generate concentration gradient of culture medium and antibiotics; chamber layer is used to culture bacteria; gas channel layer is used to remove gas in the chambers during the injection of the bacteria solution. The chip is 75 mm × 25 mm in size. (**b**) Schematic platform of culture and detection process: it consists of an inverted microscope, a data acquisition module, two three-way valves and a light-emitting diode (LED) lamp. (**c**) One gas channel array consisting of 25 microchannels with 15 μm in width and 5 μm in depth. (**d**) The flanged chamber for bacterial culture.

The microfluidic device (Figure 1a) consisted of a channel layer, a chamber layer, and a gas channel layer from top to bottom. The photograph of the microfluidic device was shown in Figure S1. The top layer and the bottom layer were made of polydimethylsiloxane (PDMS), and the middle layer was made of glass. The middle layer was 75 mm × 25 mm × 1.2 mm in dimension and consisted of 36 1-mm-diameter holes as bacteria culture chambers. The honeycomb-structure microchannels for generating concentration gradient of culture medium and antibiotics solutions were fabricated on the bottom of the top PDMS layer using standard soft lithography techniques (Illustrated in the Supplementary Information). Each channel of the honeycomb structure was 400 μm in width, 30 μm in depth, and 180 μm in length. To improve the mixing of the solutions, a micropillar array was fabricated in each vertical channel of the honeycomb structure. The top view of the micropillar was parallelogram, the sizes of which are 100 μm and 70 μm long, respectively, and the acute angle of which was 45 degrees. The space in horizontal direction between two neighbored micropillars was 50 μm, and the angle of the array was 45 degrees to the vertical direction. The diameter of the channel connected to the chamber in the top layer was 2 mm. The bottom layer was composed of 36 arrays of gas channels, which were used to remove the gas in the chambers during the injection of the bacteria solution. Each gas channel array (Figure 1c) contained 25 microchannels with 15 μm in width and 5 μm in depth.

### 2.2. Instrumentation

The experimental setup shown in Figure 1b consisted of an inverted microscope (Olympus, IX71, Tokyo, Japan), a data acquisition module (DAQ, National Instruments, USB-6229, Austin, TX, USA), two three-way valves (Labsmith, Livermore, CA, USA), and four syringe pumps (WPI, AL-1000, Sarasota, FL, USA). A 490-nm LED lamp (Thorlabs, M490L4, Newton, NJ, USA) was used as the incident light source. The light-emitting diode (LED) brightness, i.e., the LED current, was precisely controlled by an external voltage from the DAQ module. The light was focused onto the chamber by a condenser, and was finally collected by a photomultiplier tube (PMT, Hamamatsu, H7422-40, Hamamatsu, Japan) after passing through a 4× objective lens. The acquisition of the PMT signal data was carried out at 100 KHz via the DAQ module using the LabVIEW software (2016, National Instruments, Austin, TX, USA)

### 2.3. Bacteria Strains and Pre-culture

*E. coli* (CGMCC 1.2385, China General Microbiological Culture Collection Center, Beijing, China) was used throughout this study. The bacteria were in the storage freeze-dried powder. Firstly, the powder was immersed in the 1 mL sterile water. The sample was plated on Luria-Bertani broth (LB broth) agar plate medium by inoculation loop. After 24 h of cultivation, the bacteria from the plate medium were transferred to LB medium consisting of 0.5% yeast extract, 1% peptone, 1% NaCl in deionized water with pH = 7.4, and incubated overnight. For the measurement experiment, an inoculation was then from the LB broth pre-culture into M9 medium (12.8 g/L sodium phosphate (dibasic) heptahydrate, 3 g/L monopotassium phosphate, 0.5 g/L sodium chloride, 1 g/L ammonium chloride, 20 mL of 20% D-Glucose solution, 2 mL of 1.0 M MgSO_4_ solution, and 0.1 mL of 1.0 M CaCl_2_ solution) for 16 h at 37 °C.

### 2.4. Bacterial Culture and Inhibitiory Test on Chip

The microchannels and the chambers were firstly sterilized by flushing with 75% alcohol overnight and sterilized deionized water (DI water) in sequence. After ultraviolet (UV) sterilization for 30 min, *E.coli* in M9 medium (optical density (OD) in the range of 0.112–0.115) was injected into the chambers at the constant flow rate of 5 μL/min using syringe pumps. The chip was then flushed with culture medium and antibiotics at 2 μL/min to wash the bacteria away from the microchannels. After balancing for 2 min, concentration gradient was generated. The optical density in each culture chamber was measured every 1 h during the bacteria culture.

### 2.5. Bacterial Culture and Inhibitiory Test off Chip

The growth kinetics of *E. coli* cultured on the chips were compared with 96-well plates. A total of 40 μL of bacterial suspension and 360 μL M9 medium with or without amoxicillin (5 mg/L, 4 mg/L, 3 mg/L, 2 mg/L, 1 mg/L) were pipetted to three replicate wells. The 96-well plates were placed on automated growth curve system (Bioscreen C, Oy Growth Curves Ab Ltd, Helsinki, Finland) and the OD values were recorded every 15 min for 35 h.

## 3. Results and Discussion

### 3.1. On-chip Culture Strategy

#### 3.1.1. Concentration Gradient Generator

**Figure 2:**
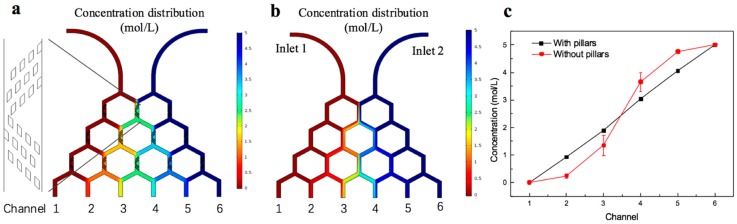
The simulation of concentration distribution in surface field. (**a**) The honey-comb structure with micropillar arrays. (**b**) The honey-comb structure without micropillar arrays. (**c**) The linearity of the concentration outflow from the 6 outlets of the structures with micropillar and without micropillar arrays. R^2^ is 0.998. The error bar is standard deviation calculated by the concentration distribution of the outlet.

In order to obtain sufficient mixing of two solutions, the lengths of vertical channels should be long enough. The Christmas tree structure has been widely used as one of the controllable and effective gradient generators. However, zig-zag mixers inside the Christmas tree structure may result in high hydraulic resistance and big area occupation [35]. To solve this issue, passive mixers and passive diffusion are used in microfluidic devices to increase mixing effects [36,37]. In this experiment, we fabricated some parallelogram micropillar arrays in the vertical channels. Molecular motion was promoted due to obstacles in flow direction, so that mixing effects were facilitated. The surface concentration field in stationary fluid static of the two structures, with and without micropillar arrays, were analyzed by COMSOL Multiphysics (5.2, COMSOL Inc, Middlesex County, MA, USA) respectively. The inlet 1 is injected by water and the inlet 2 is injected by 5 mol/L glucose solution. Simulation result shows the mixture of each outlet is more uniform in the generator with micropillar arrays (Figure 2a). Nevertheless, the diffusion in the generator without micropillar arrays was not sufficient because of short channels. The concentration encounter was used to describe concentration range of each outlet, which is shown in Figures S2 and S3. In addition, we calculated the average value of each outlet and contrasted both structures of the concentration distribution in outlet. The generator with micropillar arrays showed a good linear relationship between the channels and the concentration (Figure 2c), however concentration gradient was not generated linearly in the generator without micropillar arrays. At the same time, the error bars in Figure 2c shows the worse distribution at the outlet 3 and outlet 4, which is in consistent with the phenomenon in Figure 2b. The boundary of two fluids at outlet 3 and outlet 4 could be clearly distinguished.

Amoxicillin and fluorescein have similar molecular weights and consequently similar diffusion characteristics, therefore the mixing effect of amoxicillin on chip can be estimated by fluorescence imaging [22]. Sodium fluorescein and deionized (DI) water was mixed by the concentration gradient generator, and fluorescence intensity of outlets were detected. The result was shown in Figure S4. 20 μmol/L sodium fluorescein and water were injected at the velocity of 2 μL/min. After balancing for 5 min, the fluorescence images were recorded by charge coupled device (CCD), which were shown in Figure S4b. The images were analyzed by Image J, and the result was shown in Figure S4c. The concentration outflow from 6 outlets are in linear, and the R^2^ is 0.989.

#### 3.1.2. Improved Flanged Chamber for Continuous-Flow Bacteria Culture

**Figure 3:**
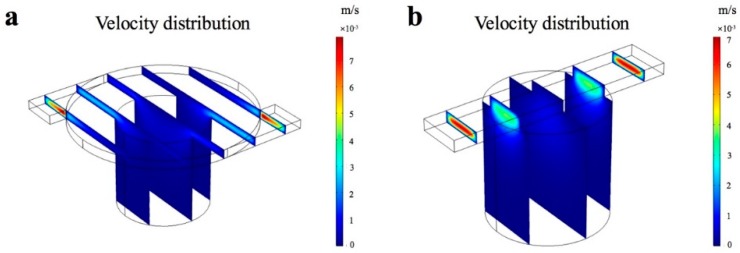
The simulation of velocity distribution in slice field. (**a**) Flanged chamber with 2 mm diameter round structure connected to the chamber. (**b**) Straight channel connected to the culture chamber.

We developed a novel flanged chamber with 2-mm-diameter round structure connected to the chamber, instead of applying a straight channel passing above it (Figure 1d). This flanged chamber is advantageous for medium diffusion and can keep the bacteria from shear force. The velocity field of both chambers in stationary fluidic static was analyzed by COMSOL Multiphysics. The result in Figure 3a indicates that the velocity distribution in the channel, which is connected to the flanged chamber, is steady when the solution passes through. However, there was high speed fluctuation of liquid flow in the straight channel (Figure 3b). Hence, the solution with larger surface area between channel and chamber can avoid the influence of flow velocity.

The bacteria were cultured on micro-scale chamber, which provided larger volume for reducing the initial concentration of bacteria. At the same time, the medium in chamber was refreshed constantly by the diffusion of continuous flow. The main part of this chip is PDMS, and the amount of oxygen dissolved in it is calculated (Illustrated in Supplementary Information). It concludes that sufficient oxygen dissolved in PDMS can keep the bacteria in good condition during the culture. Therefore, the continuous flow not only supplies the fresh oxygen and nutrient to bacteria but also removes metabolic waste, ensuring that the bacteria is able to exponentially grow. The diffusion constant (*D*) of a small molecule like glucose is about 3 × 10^−6^ cm^2^/s to 10 × 10^−6^ cm^2^/s at low concentration [38]. As the carbon sources of M9 medium, glucose diffusion time (*t*) is estimated from 1000 s to 3333 s between the channel and culture chamber according to the diffusion equation, *t = L^2^/D*. The diffusion process of solution between chamber and channel was shown in Supplementary Figure S5. The diffusion time of orange solution was consistent with theoretical calculation. After loading the orange solution, the 0.4% glucose solution with a velocity of 2 μL/min was injected. The image of color solution diffusion at different time was taken by a CCD camera, and the color intensity of solution in chamber was analyzed by Image J (Figure S6). With the color solution in channel flowed away, the intensity decreased mildly in the first 5 min, then descend sharply at 5th min. The result was also shown in supplementary movie, the color solution obviously faded after 11 min. The pale yellow solution emerged and the solution was almost colorless at 38 min. It implies that the medium solution in culture chamber was exchanged completely every 38 min, which was close to the generation time of bacteria. It has been reported that it generally takes 20–30 min for bacterial cell division [14]. Compared to traditional bacteria culture, the continuous flow of this experiment is able to supply fresh medium for almost each generation culture of bacteria.

#### 3.1.3. Function of Gas Channels 

**Figure 4:**
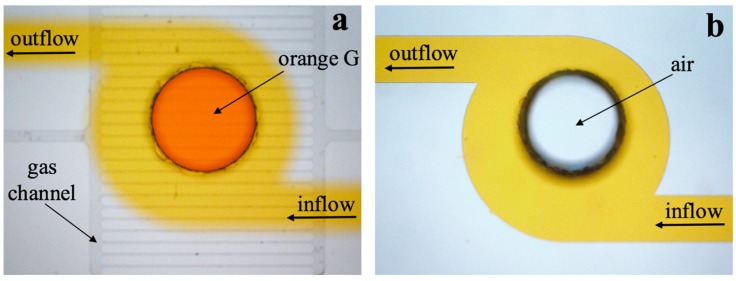
Characterization of orange solution injecting process and the function of gas channel. (**a**) Liquid loading into the chamber with the array of gas channel. Orange G was easily loaded into culture chamber. (**b**) Liquid failed to be loaded into the chamber without gas channel. The culture chamber was empty, and orange G passed through the channel.

The chambers are used to trap and culture bacteria. For the purpose of protecting bacteria from shear force, the larger and deeper chambers are better [39,40]. However, it is difficult to load the bacteria into chambers. The surface tension of liquid is hard to break due to laminar flow, therefore, the air in chamber cannot be pumped off by sufficient stress, resulting in the failure of loading the sample into chambers. One gas channel array consisting of 25 microchannels with 15 μm in width and 5 μm in depth was designed to exhaust the air in culture chamber. Figure 4a shows that the liquid can be flowed into the chamber with gas channel, while it failed to load into the chamber without gas channel, shown in Figure 4b. The main gas channel linking to the air is closed after sample loading, and there is no orange G solution in it (Figure 4a).

### 3.2. Assessment of On-chip Bacterial Culture 

For on-chip experiment, medium M9 was used to culture *E.coli* in chip and in well plate. It can provide minimum salt conditions required for bacterial growth, and act as a filter against contaminations by microorganisms that need additives such as amino acids [41]. In addition, compared to the rich growth medium, it is simple to ensure the clear signal detected by PMT. In this study, the turbidity of medium increases with the bacterial growth, and the transmittance is monitored by PMT.

**Figure 5:**
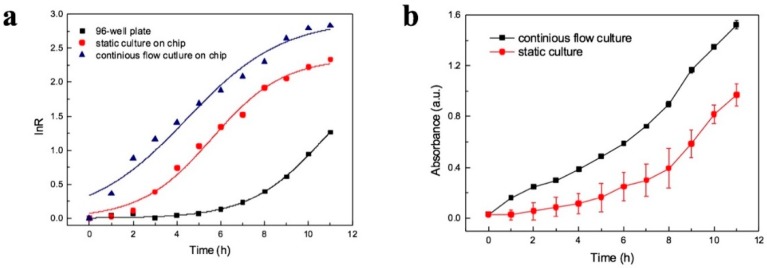
Bacterial growth curve fittings (**a**) 96-well plate, static culture on chip and continuous flow culture on chip (*R* = optical density (*OD)*/*OD*_0_). (**b**) On chip culture in two methods: continuous flow culture and static culture on chip. The error bar is obtained by three of six culture chambers at the same concentration.

Relative growth rate has been widely used to quantify the speed of bacterial growth. In this study, we compare relative growth rate *μ* (h^−1^) on chip and in 96-well plate to assess both approaches of bacterial growth. In general, relative growth rate depends on the ration of ln(*OD/OD_0_*) and time (*μt =* ln(*OD/OD_0_*)), and *OD/OD_0_* is the increment ratio, represented by R. Figure 5a shows the bacterial growth curve fittings of 96-well plate, static culture on chip and continuous flow culture on chip. Logistic growth model has been applied to the curves, and the corresponding R^2^ is 0.995, 0.967, 0.989. The details of model and fitting data are in Supplementary Information. In traditional 96-well plate culture, the max relative growth rate is 0.29 h^−1^, which is lower than continuous flow culture on chip (*μ*_max_ = 0.36). The lag time is 6.58 h by the method of 96-well plate, while in comparison, it is only 0.06 h for on-chip culture. It indicates that bacteria grow more rapidly in micro-scale environment. This is also due to bacterial culture on chip in continuous flow which refresh the medium of chamber and supply the sufficient nutrient to bacteria. At the same time, the continuous flow can take away metabolic toxin or waste products.

The growth of *E.coli* in batch culture and continuous flow culture were observed on chip for 11 h. In static culture, the M9 medium was stopped to flow after loading the bacteria into the chamber. The result is shown in Figure 5b. The *μ*_max_ of static culture on chip is 0.343, which is lower than continuous flow culture on chip. What need to be noted is that during the period of bacterial growth in batch culture, there were several bubbles forming in the culture chamber. It may be caused by the gas produced in the process of metabolism of bacterial growth. Micro-size bubbles were formed in side wall of culture chamber during the process of bacterial growth in batch culture, which leads to error bars larger than in continuous flow (Figure 5b). In comparison, no bubbles were observed during bacterial growth on chip in continuous flow culture for up to 11 h. This implies that continuous flow is capable of taking away small amount of gas. In addition, the growth rate of bacteria in continuous flow is faster than without continuous flow and 96-well plate. It is possible that bacteria accumulate in the area with abundant nutrient in the effect of chemotaxis, and they are more likely to acclimatize by communicating with each other. Therefore, continuous flow can promote bacteria to acclimatize the new environment and grow. 

### 3.3. The Inhibition Test on Chip and Well Plate

**Figure 6:**
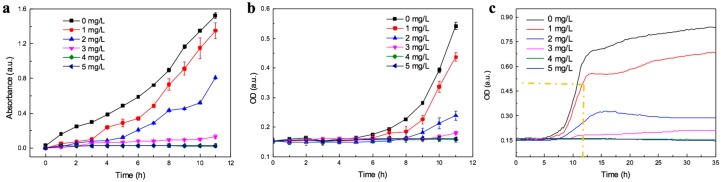
Inhibition test of *E. coli* by amoxicillin: (**a**) The growth curve of bacteria on chip exposed to different concentrations of amoxicillin for 11 h. (**b**) Bacterial growth in 96-well plate exposed to different concentrations of amoxicillin for first 11 h. (**c**) Bacterial growth in 96-well plate for 35 h. Bacterial growth for first 11 h is marked by yellow dash line.

On the basis of the study above, *E.coli* were exposed to amoxicillin for inhibition test. The microfluidic platform was applied to quantitatively analyze the inhibition of antibiotic amoxicillin on *E.coli*. After injecting *E.coli* into the culture chamber, M9 medium and 5 mg/L amoxicillin were simultaneously loaded into the channel at 2 μL/min, then the concentration gradient was generated. Figure 6a shows the curves of bacterial growth in different amoxicillin concentrations, and the error bar is calculated by three of six culture chambers at the same concentration. This implies that the array of culture chambers could be used as parallel experiments. In long-term observation, the bacteria grow rapidly in control group (without antibiotic), however, lag phase is enlarged with the antibiotic concentration increasing. At 1 mg/L and 2 mg/L amoxicillin, the bacteria respectively begin to grow after 2.59 h and 3.79 h of incubation, which is obtained by the fitting curve of bacterial growth (in Figure S7a) and the parameters in Table S1. At 3 mg/L amoxicillin, the growth curve is mild; and at 4 mg/L and 5 mg/L, the curve shows almost zero growth. Amoxicillin is one of β-lactam antibiotics, and it inhibits microbial division by preventing the synthesis of peptidoglycan, which is the most important component of bacterial cell wall. With the penicillin binding proteins (PBPs) catalyst, the peptidoglycan is cross linked by glycan strands of the polymer [42]. β-lactam antibiotics bind PBPs easily with the similar in chemical structure, then inactivates the PBPs. The bacterial cell wall is ruptured for the lack of peptidoglycan and the bacteria failed to divide. In the end, the bacteria stop to grow, whereas it is hardly to lysis under the osmoprotective conditions in the lab, which are often hypotonic [43]. Therefore, it is difficult to observe the decline of bacteria growth by *OD*. 

The inhibition test on 96-well plate within 11 hours is shown in Figure 6b. The bacterial growth is not in stationary phase, so the inhibition test in 96-well plate need longer time. Figure 6c shows bacterial growth with different concentrations of amoxicillin for 35 h. In 96-well plate, the bacterial growth curve of 3 mg/L begins to increase at 10 h, and the bacteria are in stationary phase from the 15th h. It is obvious that it will take at least 15 h to obtain the inhibition concentration. Therefore, the MIC can be obtained within 15 h by 96-well plate. In comparison, the inhibition could be obviously observed from the 6th h on chip, so the result of inhibition test on chip can be acquired within 6 h. Comparison experiments illustrate that under the condition of continuous flow on chip, the rate of bacterial growth and acquisition of MIC value is faster.

**Figure 7:**
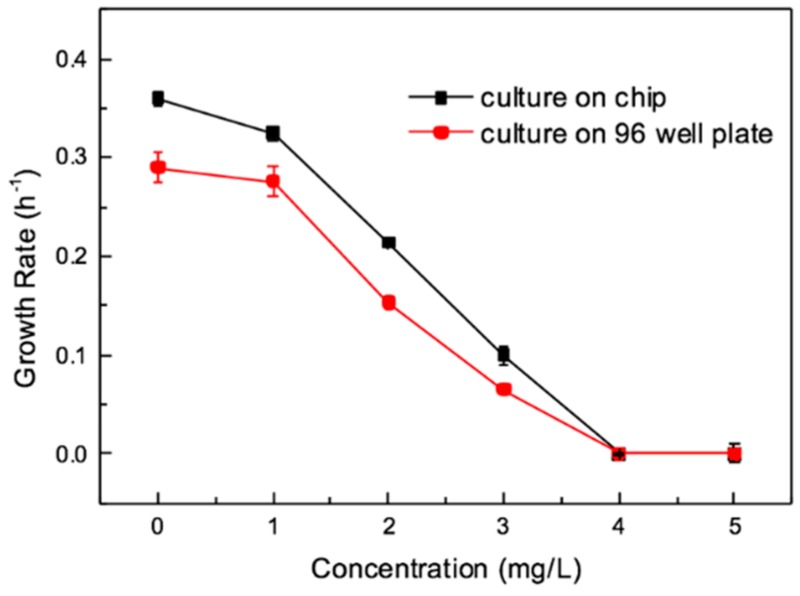
Concentration and growth rate curves for on-chip and well plate test. Error bars represent standard deviations (n = 3).

Figure 7 is the curve of concentration and growth rates for on chip and well plate. The max growth rates were obtained from the fitting curves, which were shown in Figure S7b (the parameters in Table S2), and biological parameters are also shown in Supplementary Information. It shows that at 3 mg/L, the bacterial growth was obviously inhibited in both chip and well plate tests, which could also be seen from growth curve. The growth rate of bacteria culture on well plate and on chip approaches to zero at antibiotic concentration above 4 mg/L. Therefore, the MIC was 4 mg/L. This result was in the range of 2–8 mg/L reported by the Clinical and Laboratory Standard Institute [44]. 

## 4. Conclusions

A developed microfluidic device for bacteria growth under the condition of continuous culture was designed and fabricated using the soft lithography method. This PDMS chip includes a submicro-liter-chamber array for bacterial culture and a concentration gradient generator by novel micromixers. Using the microfluidic flanged chamber, the bacterial sample was easily loaded into the chip and the amoxicillin concentration gradient was generated. The bacteria were cultured on chip under the condition of continuous flow, which could supply the sufficient oxygen and nutrient for bacteria growth in exponential phase. In addition, the flanged chamber can keep bacteria from shear force and the low concentration of initial bacteria is needed. Compare to the 96-well plate test, the growth rate is rapid on chip, and the bacterial growth with antibiotic inhibition can be quantitatively measured. Therefore, the developed microfluidic device may be a prospect to fast screen bacteria and rapid MIC test.

## Supplementary Materials

The following are available online at https://www.mdpi.com/2072-666X/11/2/207/s1, Figure S1: The photograph of the microfluidic device, Figure S2: Simulation results of concentration distribution in six channels of Figure 2a, Figure S3: Simulation results of concentration distribution in six channels of Figure 2b, Figure S4: The characterization of concentration gradient in the chip using sodium fluorescein, Figure S5: the exchange process of orange solution in reservoir and channel, Figure S6: Analysis of color intensity of solution in reservoir changed at different time, Figure S7: (a) The fitting curve of bacterial culture on chip at different concentrations of amoxicillin. (b) The fitting curve of bacterial culture on 96-well plate at different concentrations of amoxicillin, Table S1: On chip culture; Table S2: 96-well chip culture.
