# Supplementary material for: On-chip MIC by Combining Concentration Gradient Generator and Flanged Chamber Arrays
Source: Micromachines (Basel). 2020 Feb 17;11(2):207. doi: 10.3390/mi11020207 (PMC7074598; doi:10.3390/mi11020207)
Supplement: Supplementary file 1 [file micromachines-11-00207-s001.pdf]

micromachines

Supplementary Information: On-chip MIC by Combining Concentration Gradient Generator and Flanged Chamber Arrays

Xiao-Yan Zhang [1], Zhe-Yu Li [1,*], Kose Ueno [2], Hiroaki Misawa [2,3] , Nan-Qi Ren [1] and Kai Sun [1,*]

1 State Key Laboratory of Urban Water Resource and Environment, School of Environment, Harbin Institute of Technology, Harbin 150090, China; xyzhang774985529@163.com (X.-Y.Z.); rnq@hit.edu.cn (N.-Q.R.)
2 Research Institute for Electronic Science, Hokkaido University, Sapporo 001-0021, Japan; k-ueno@es.hokudai.ac.jp
3 Department of Applied Chemistry & Institute of Molecular Science, National Chiao Tung University, Hsinchu 30010, Taiwan; misawa@es.hokudai.ac.jp
* Correspondence: ksun@hit.edu.cn (K.S.); zhylee@hit.edu.cn (Z.-Y.L.); Tel.: +86-451-8628-9126 (K.S.); +86-451-8628-6808 (Z.-Y.L.)

Received: 21 January 2020; Accepted: 14 February 2020; Published: date

Microfluidic Chip Preparation

The bottom and top layer were prepared by standard soft lithographic process. The middle layer was borosilicate glass with 36 holes punched by laser, and the culture chamber was 1.2 mm deep, 1 mm diameter. SU-8 2015 and 2050 (Microchem Corp., Newton, MA, USA) were respectively molded to fabricate the bottom layer and top layer. A 10:1 mixer of PDMS (Sylgard 184, Dow Corning, Midland, MI, USA) prepolymer and curing agent were poured onto SU-8 mold after degassing. After baking at 70 °C in oven, the PDMS mold was peeled off and used to bond with glass.

The borosilicate glass with 36 holes was cleaned in organic and metallic contamination in sequence for 5 min in following solutions, H_2SO_4:H_2O_2 (4:1), H_2O: H_2O_2: NH_4OH (50:10:1), H_2O:H_2O_2: HCl (5:1:1). After washing the substrate, a $H_2O/H_2O_2/NH_4OH$ solution (7:2:1) was used to increase the hydrophilicity of the glass plates. The top layer PDMS and the middle layer glass were bounded to each other by using UV o-zone cleaner (Jelight) for 5 min and baking for 70 °C for 15 min. The bottom layer was reversibly adhered to the upper two layers.

In the chamber layer, the diameter of each culture chamber is 1 mm. On the gas channel layer, the length of each side of the gas channel array is 2.8 mm, which is much larger than the culture chamber. The alignment of the two layers is quite easy. Although the diameter of the flanged channel on top of the chamber is 2 mm, which is also larger than the size of the chamber, comparing to the above alignment, it is a little difficult to handle the top layer and the middle layer. The fabricated chips will be checked by the microscope. Some shift of the alignment could be ignored (as shown in Figure 4). The chip with bad alignment cannot be used. Anyway, the fabrication is only suitable for the laboratory.

The Gas Permeation

PDMS is used as the main part of this chip, because it is transparent and gas permeable material, with the performance of gas sorption, diffusion, and permeation, which follow Henry's Law [1,2]. It has a high oxygen diffusivity, which is necessary for bacterial growth [3], and the amount of maximum oxygen Fmax diffusing through the PDMSs can be estimated by the following diffusion equation [4]:

$$F\max \approx D_{PDMS} (\triangle C/\triangle z)$$

D_{PDMS}, the diffusivity of oxygen in PDMS is 4.1×10^{-9} m²/s. $\triangle C$, the oxygen concentration gradient across the PDMS layer is 0.2 mol O^2/m^3. $\triangle z$ denotes the thickness of PDMS, which is 5 mm in this design. Fmax is approximated 1.29×10^{-13} mol/s. On the other hand, it is reported that the oxygen consumption rate of *E.coli* is 1.78×10^{-18} mol/cell/s. According to this value, the amount of oxygen in

one culture chamber can supply 10^5 cells *E.coli* to grow. In general, the number of bacteria in one culture chamber with the volume of 1 μL is less than 10^4 cells. Therefore, sufficient oxygen dissolved in PDMS can keep the bacteria in good condition during the culture.

Modeling of bacterial growth curve

We applied Logistic model to bacterial growth curve. The equation:

$$y = \frac{\mathrm{a}}{[1 + \exp(b - cx)]}$$

The modified equation [5] is:

$$y = \frac{\mathrm{A}}{\left\{1 + \exp\left[\frac{4\mu_m}{A}(\lambda - \mathrm{t}) + 2\right]\right\}}$$

A: asymptote,

μ_m: maximum specific growth rate (h^{-1}),

λ: lag time (t).

Reference

1. Singh, A.; Freeman, B.D.; Pinnau, I. Pure and mixed gas acetone/nitrogen permeation properties of polydimethylsiloxane [PDMS]. *J. Polym. Sci. B: Polym. Phys.* **1998**, *36*, 289–301.
2. Merkel, T.C.; Bondar, V.I.; Nagai, K.; Freeman, B.D.; Pinnau, I. Gas sorption, diffusion, and permeation in poly (dimethylsiloxane). *J. Polym. Sci. B: Polym. Phys.* **2000**, *38*, 415–434.
3. Charati, S.G.; Stern, S.A. Diffusion of gases in silicone polymers: molecular dynamics simulations. *Macromolecules* **1998**, *31*, 5529–5535.
4. Leclerc, E.; Sakai, Y.; Fujii, T. Cell culture in 3-dimensional microfluidic structure of PDMS (polydimethylsiloxane). *Biomed. Microdevices* **2003**, *5*, 109–114.
5. Zwietering, M.H.; Jongenburger, I.; Rombouts, F.M.; Van't Riet, K. Modeling of the bacterial growth curve. *Appl. Environ. Microbio.* **1990**, *56*, 1875–1881.

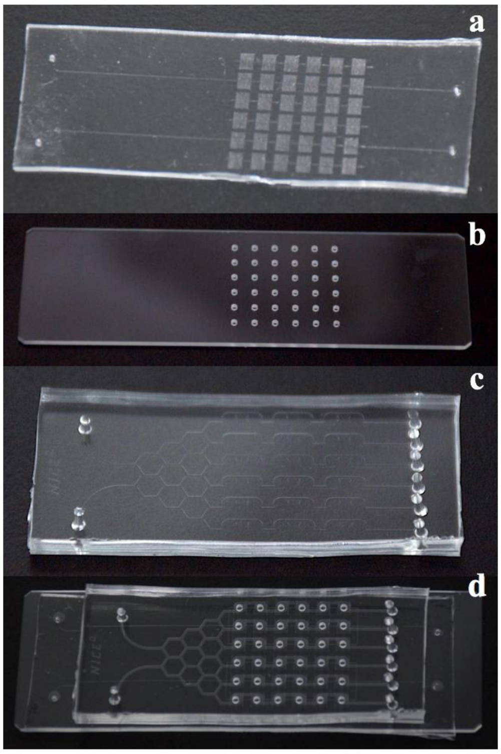

Figure S1. The photograph of the microfluidic device. (**a**) The bottom layer for removing gas in the chambers. (**b**) The middle layer for culturing bacteria. (**c**) The top layer for generating concentration gradient of culture medium and antibiotics. (**d**) The chip after bonding.

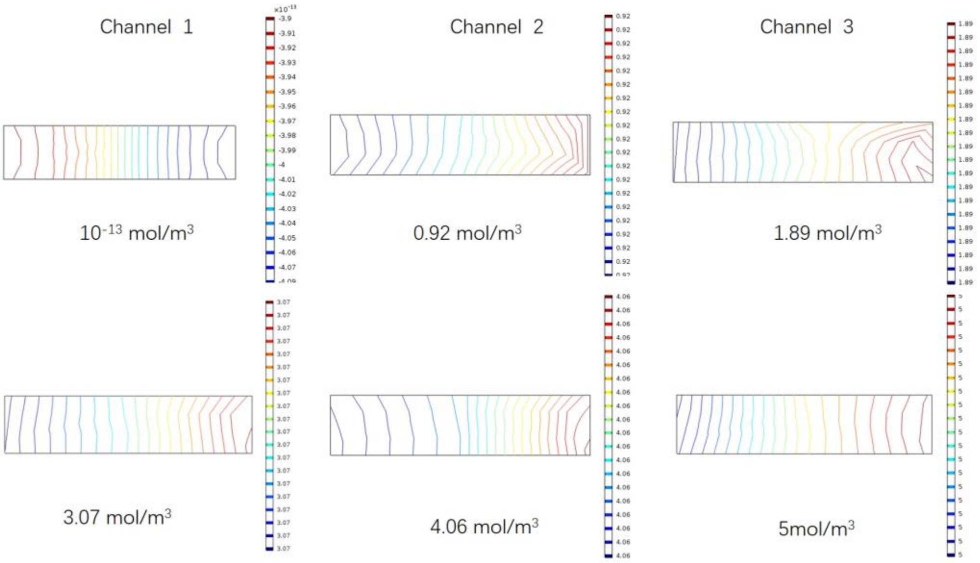

Figure S2. Simulation results of concentration distribution in six channels of Figure 2a.

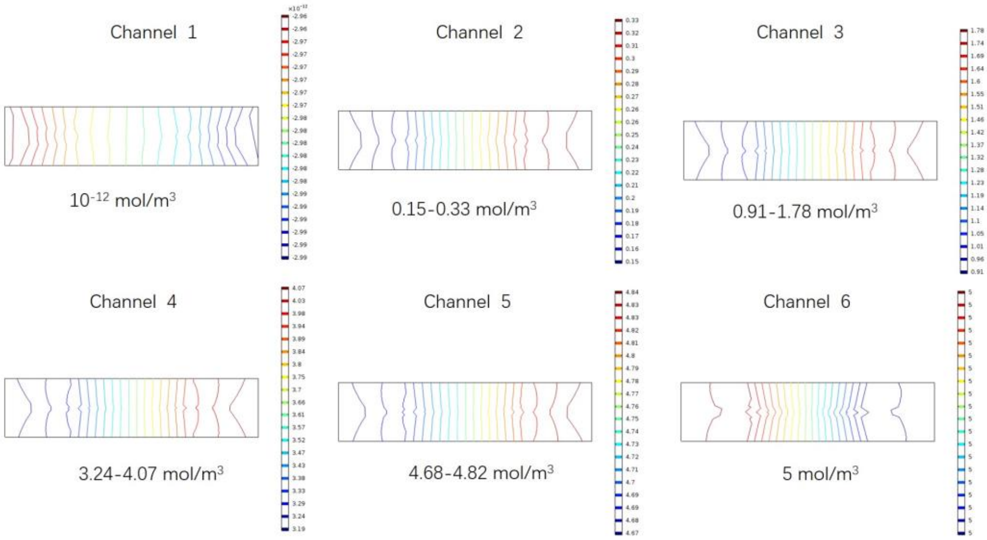

Figure S3. Simulation results of concentration distribution in six channels of Figure 2b.

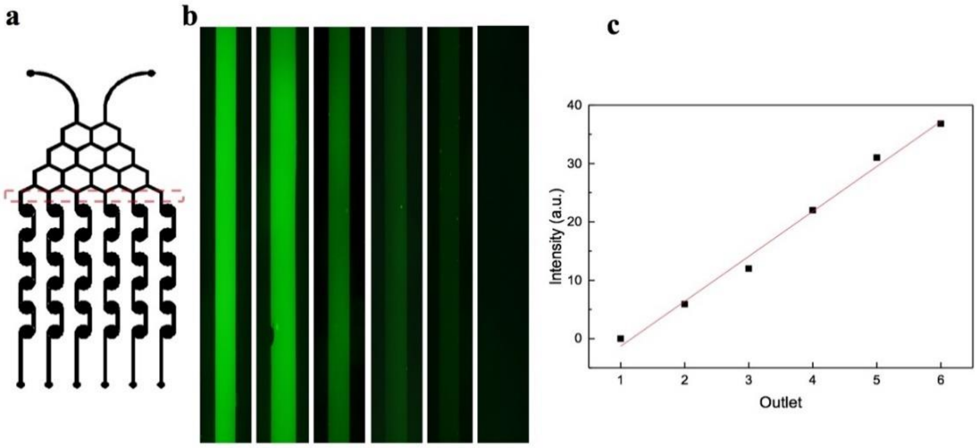

Figure S4. The characterization of concentration gradient in the chip using sodium fluorescein. (**a**) The regions of imaging. (**b**) The images of fluorescein sodium. (**c**) The linear fitting of different concentrations from the 6 outlets.

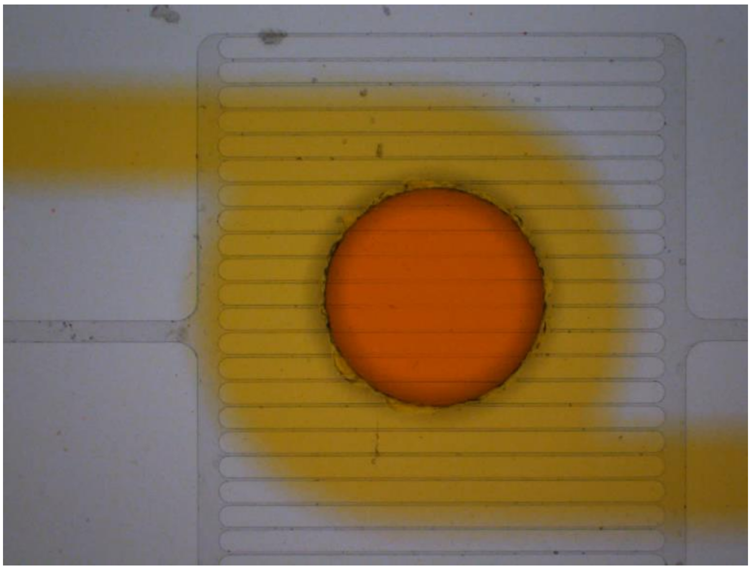

Figure S5. The exchange process of orange solution in reservoir and channel.

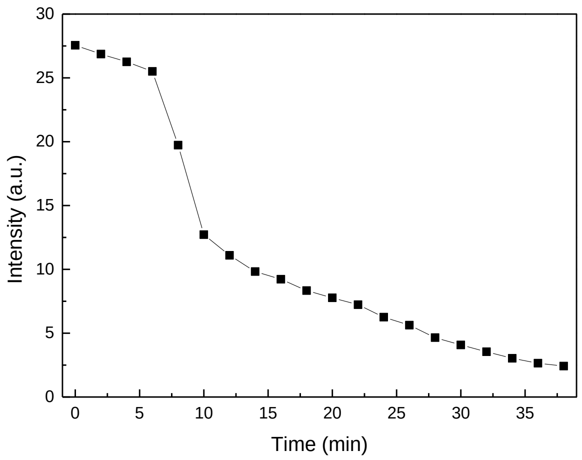

Figure S6. Analysis of color intensity of solution in reservoir changed at different time.

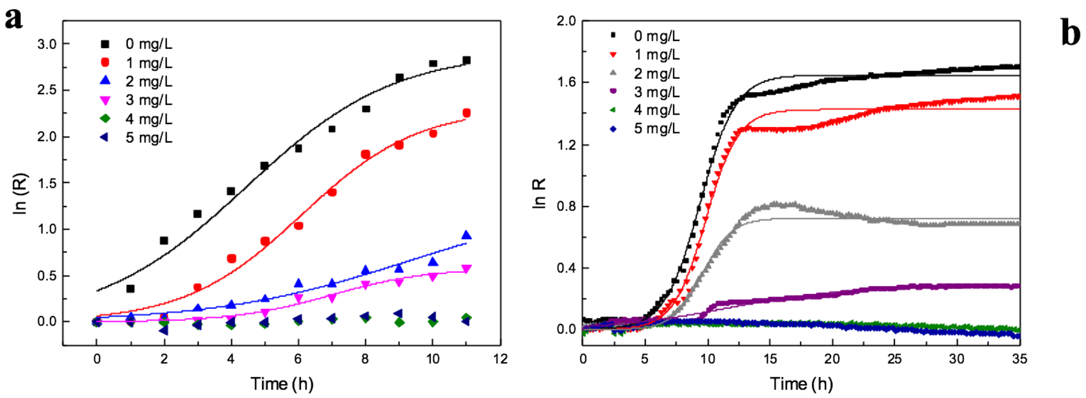

Figure S7. (**a**) The fitting curve of bacterial culture on chip at different concentrations of amoxicillin. (**b**) The fitting curve of bacterial culture on 96-well plate at different concentrations of amoxicillin.

Table S1. On chip culture.

Concentration	A	μ_m (h^{-1})	λ (t)	R^2
0 mg/L	1.65	0.29	6.58	0.995
1 mg/L	1.427	0.276	7.271	0.988
2 mg/L	0.723	0.153	7.21	0.98
3 mg/L	0.2785	0.1048	7.39	0.969
4 mg/L	–	0	–	–
5 mg/L	–	0	–	–

Table S2. 96-well chip culture.

Concentration	A	μ_m	λ	R^2
0 mg/L	2.92	0.36	0.06	0.989
1 mg/L	2.327	0.325	2.589	0.984
2 mg/L	1.475	0.14	3.59	0.958
3 mg/L	0.582	0.1	4.08	0.976
4 mg/L	–	0	–	–
5 mg/L	–	0	–	–
